# Laser-Induced Graphene-Based Enzymatic Biosensor for Glucose Detection

**DOI:** 10.3390/polym13162795

**Published:** 2021-08-20

**Authors:** Kalpana Settu, Pin-Tzu Chiu, Yu-Ming Huang

**Affiliations:** Department of Electrical Engineering, National Taipei University, New Taipei City 23741, Taiwan; s410787003@gm.ntpu.edu.tw (P.-T.C.); idris.herondale@gmail.com (Y.-M.H.)

**Keywords:** biosensor, laser-induced graphene, polyimide, glucose, enzyme

## Abstract

Laser-induced graphene (LIG) has recently been receiving increasing attention due to its simple fabrication and low cost. This study reports a flexible laser-induced graphene-based electrochemical biosensor fabricated on a polymer substrate by the laser direct engraving process. For this purpose, a 450 nm UV laser was employed to produce a laser-induced graphene electrode (LIGE) on a polyimide substrate. After the laser engraving of LIGE, the chitosan–glucose oxidase (GOx) composite was immobilized on the LIGE surface to develop the biosensor for glucose detection. It was observed that the developed LIGE biosensor exhibited good amperometric responses toward glucose detection over a wide linear range up to 8 mM. The GOx/chitosan-modified LIGE biosensor showed high sensitivity of 43.15 µA mM^−1^ cm^−2^ with a detection limit of 0.431 mM. The interference studies performed with some possible interfering compounds such as ascorbic acid, uric acid, and urea exhibited no interference as there was no difference observed in the amperometric glucose detection. It was suggested that the LIGE-based biosensor proposed herein was easy to prepare and could be used for low-cost, rapid, and sensitive/selective glucose detection.

## 1. Introduction

Numerous novel and cutting-edge technologies and materials are necessary to satisfy the new trends and requisites of analytical systems as the needs for environmental, biomedical, food and beverage analysis are progressing very quickly. The development of biosensors has evolved as one of the most promising research directions to overcome these challenges. Therefore, biosensor-based techniques have recently started being applied for the determination of different clinically, environmentally and biologically active materials [1,2,3]. In this regard, the design of biosensors in nanoscience/nanotechnology, environmental, medicine and food monitoring has been significantly increased during the past decade for their extensive applications. These advanced technologies have assisted the construction of highly sensitive, selective, customizable, and portable sensors for the determination of various clinically significant materials such as glucose, etc. [4]. The progress of such glucose biosensors has an inordinate significance in diagnosing and controlling diabetes mellitus, which is considered a worldwide public health problem. Diabetes mellitus would increase the risk of heart disease, kidney failure, blindness, postoperative and wound infections [5,6].

Diabetes mellitus has increased worldwide over the past five decades. Diabetes is a medical condition in which patients experience glucose concentration diverging from the normal range of 80–120 mg/dL (4.4–6.6 mM) [7]. In 2019, the International Diabetes Federation (IDF) assessments indicated that approximately 463 million adults have diabetes, and it might rise to 700 million by 2045 [8]. Diabetic patients are required to perform glucose testing several times a day to maintain normal glucose levels. Hence, the rapid quantification of glucose concentration in bodily fluids is vital for diagnosing and treating diabetic patients. For this purpose, the design of an easy, rapid and low-cost technology for the determination of glucose is essential in clinical diagnosis [9,10].

Glucose biosensors have significantly contributed to the detection of glucose levels in diabetic patients [11,12]. Studies have indicated that among various biosensors, glucose oxidase (GOx) enzyme-based electrochemical biosensors were considered to offer good selectivity and sensitivity for glucose detection [13,14]. Amperometry is the widely used electrochemical technique for glucose detection. Amperometric sensors could provide several advantages, such as ease of use, short analysis time, high sensitivity, and higher signal-to-noise ratio compared to other sensors [14,15,16]. The common idea applied for the development of amperometric biosensors is the efficiency of charge transfer, which can be better enhanced. Additionally, the biocompatibility issues of the sensors could be resolved by modifying electrodes with polymers such as chitosan or hydrogels [17]. In addition, various features of the electrodes could easily be altered by selecting the optimal chemical and electrochemical parameters during the effective electrode modifications [18,19].

The amperometric glucose biosensor generally uses an enzyme glucose oxidase (GOx), which catalyzes glucose oxidation at the electrode and provides high selectivity in glucose detection. Most enzymatic amperometric biosensors are based on disposable screen-printed enzyme electrode strips [20,21,22]. However, the wastage of materials might occur during the screen-printing process, limiting the applications of screen-printed electrodes.

Graphene, a carbon-based nanomaterial, has gained substantial attention in many areas. In terms of electrochemical properties, graphene could provide high conductivity with a remarkable heterogeneous electron transfer rate [23,24]. In 2014, it was found that polymers such as polyimide (PI) could be directly converted into porous graphene using a CO_2_ laser machine with a 10.6 μm wavelength [25]. In addition to infrared CO_2_ (10.6 μm) laser, visible laser [26,27,28,29,30,31] and ultraviolet laser [32] have also been successfully used to synthesize laser-induced graphene (LIG). The laser-irradiation of the PI film caused the photo-thermal generation of the graphene due to the local heating of the film. Upon heating the film, the carbon atoms bonded with oxygen (C–O, C=O) and nitrogen (C–N) atoms via sp^3^ and sp^2^ hybridization breakdown and rearranged to form several layers of sp^2^ hybridized carbon atoms of graphene [25,33]. The laser induction of graphene has been performed in ambient conditions without any material wastage. In addition, the shape/pattern of LIG could also be easily customized by computer design, which holds great promise toward developing glucose biosensors.

Recently, Pereira et al. demonstrated the electrochemical response of GOx adsorbed on a CO_2_ laser-scribed LIG [34]. The GOx enzyme adsorbed on LIG remained catalytically active even after running the cyclic voltammetry up to +1.0 V for glucose detection. The LIG electrodes facilitated the direct electron transfer between the GOx and the electrode surface without mediators.

In this study, we fabricated a laser-induced graphene electrode (LIGE) by simple direct laser engraving with the UV laser on polyimide tape. The LIGE surface was immobilized with GOx/Chitosan composite for selective detection on glucose. Amperometric measurement was used to quantify the glucose concentration with the developed LIGE enzymatic biosensor. The novelty of the present work lies in the detection of glucose with enhanced sensitivity using a simple, low-cost LIGE-based biosensor.

## 2. Materials and Methods

### 2.1. Chemicals and Instruments

Glucose, uric acid, ascorbic acid, chitosan, and glucose oxidase (GOx, from Aspergillus niger, Type X-S, lyophilized powder, 118,000 units/g solid) were purchased from Sigma-Aldrich Corp. (St. Louis, MO, USA). A single-sided Kapton^®^ polyimide tape with a film thickness of ~30.4 μm and a width of 50 mm was obtained from STAREK Scientific Co., Ltd. (Taipei, Taiwan). Photo/printing paper (HYA300, A4—120 gm^−2^, 0.15 mm) was purchased from a local book store. All the electrochemical measurements were conducted using a portable potentiostat (PalmSens 4, PalmSens, Houten, The Netherlands). Raman spectroscopic study was conducted using a micro-Raman spectrometer (JASCO NRS-4100; Laser 532 nm) with a spectral resolution of 2 cm^−1^. Data processing/plotting was performed using Origin 9.1 software (OriginLab Inc., Northampton, MA, USA).

### 2.2. Fabrication of LIGE Sensor

**Figure 1:**
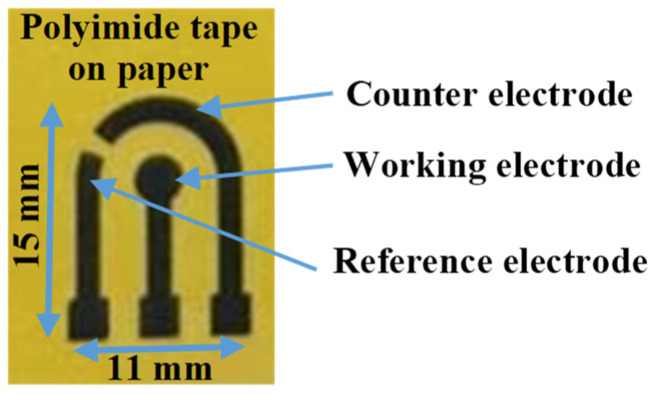
LIG 3-electrode system on polyimide tape fabricated by laser inscribing.

A 3-electrode system was designed using AutoCAD software with a 3 mm diameter of working electrode and laser-inscribed to graphene-based electrodes. Kapton^®^ polyimide tape was pasted onto a paper substrate and cleaned with isopropanol and deionized water. Then, the designed pattern made in graphic software was inscribed on the surface of the Kapton tape using a laser engraving machine (HANLIN 7WLS, 7 W, 450 nm) to form highly conductive graphene electrodes, as shown in Figure 1. The resistance of the graphene-based electrode was optimized by adjusting the laser power intensity (22% of the machine’s maximum power), engraving depth (5%), the distance between the laser head and the polyimide substrate (~13 cm). The duration for fabricating a complete LIGE sensor was 2.8 min.

### 2.3. Immobilization of GOx/Chitosan Composite on the LIGE

The glucose biosensor was prepared by immobilizing the glucose oxidase and chitosan hydrogel homogeneous biocomposite on the LIGE surface. The resulting biocomposite could retain the enzyme bioactivity at considerably extreme conditions [35]. Five milligrams of GOx and three milligrams of chitosan were dissolved in 0.5 mL of deionized water and stirred for 5 min [36]. Subsequently, 5 μL of the mixture was cast onto the surface of the LIGE working electrode. Then, the LIGE sensor was kept in a refrigerator at 4 °C for 24 h.

### 2.4. Electrochemical Measurements

All the electrochemical measurements were carried out using PalmSens 4 potentiostat (PalmSens, Houten, The Netherlands) at room temperature. The electrochemical redox characteristics of the LIGE were measured by Cyclic voltammetry (CV) with different concentrations of potassium ferri (III)cyanide (K_3_[Fe(CN)_6_]) in 50 mM of phosphate-buffered solution (PBS). CV measurements were performed at a scan rate of 50 mV/s with a potential range from −0.8 to +0.8 V. Chronoamperometry (CA) experiments for glucose detection with LIGE were performed in 50 mM PBS at the fixed applied voltage of 0.8 V for 60 s. The detection principle of glucose is based on the electron transfer mechanism. GOx reacts with glucose in the presence of O_2_ and produces gluconolactone and H_2_O_2_. A change in electrical current occurs at the electrode surface during these reactions due to the electron transfer. Additionally, the resulting current response is proportional to the number of glucose molecules present in the sample.

### 2.5. Optimization of Applied Potential and pH

CA measurements were used to determine the optimal applied potential and pH for glucose detection. The CA potential was optimized by varying the potential from 0.3 V to 1.3 V (5 mM Glucose, pH 7), and the resulting CA current was sampled at 60 s. CA measurements were performed with a LIGE biosensor at 5 mM glucose solution with the applied potential of 0.8 V by varying the pH of the phosphate-buffered solution from a pH of 5 to 9, and the optimal pH was found.

### 2.6. Interference Study

The response of the LIGE biosensor for glucose detection was evaluated in the presence of potential interferences such as 0.1 mM ascorbic acid, 0.1 mM uric acid, and 3 mM urea (pH 7; 5 mM glucose; 0.8 V).

## 3. Results and Discussion

### 3.1. Characterization of LIGE

#### 3.1.1. Raman Spectra

**Figure 2:**
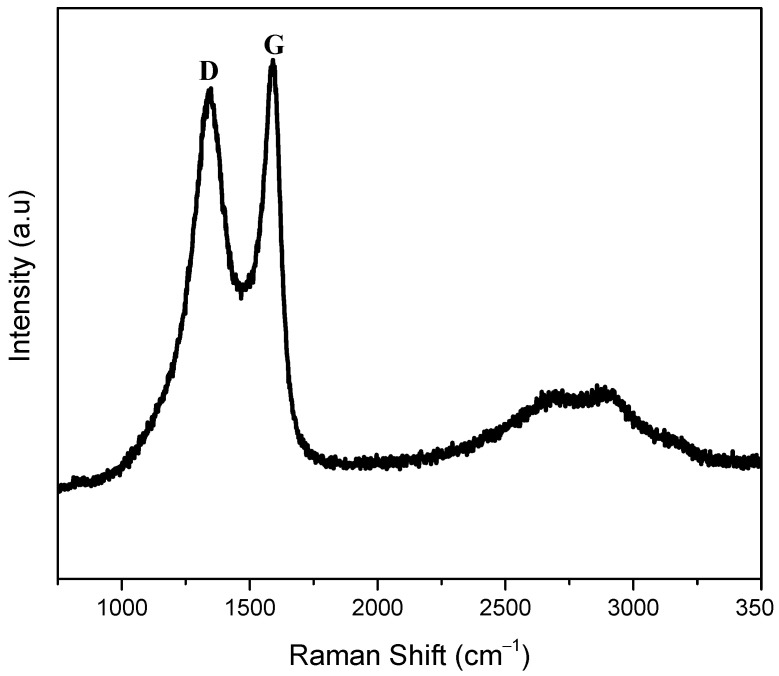
Raman spectra of LIGE.

In this study, a graphene three-electrode system for electrochemical sensing applications was developed by direct laser inscribing on polymer substrate (Polyimide). The prepared LIGE was characterized with Raman spectra, as shown in Figure 2. The Raman spectrum consists of G band at ca. 1592 cm^−1^ related to the E_2g_ phonon of the sp^2^ carbon atoms, and D band at ca. 1340 cm^−1^ corresponds to the disordered grain boundaries [37,38]. Two other bands were observed at 2697 and 2900 cm^−1^. The band at ca. 2700 cm^−1^ is known as the 2D band, an indicator of the number of graphene layers. A sharp peak will appear at ca. 2700 cm^−1^ for monolayer graphene. Here, the broadened band was observed, which would be attributed to the prepared graphene containing many layers with some defects. The band that appeared at 2900 cm^−1^ is called an S3 band, which is a second-order peak derived from the D–G peak combination. The band intensity ratio of S3–2D is proportional to the reduction in defects [38]. This Raman spectra result indicated that the obtained black material on polyimide substrate was carbon-based graphene.

#### 3.1.2. Electrochemical Characterization

**Figure 3:**
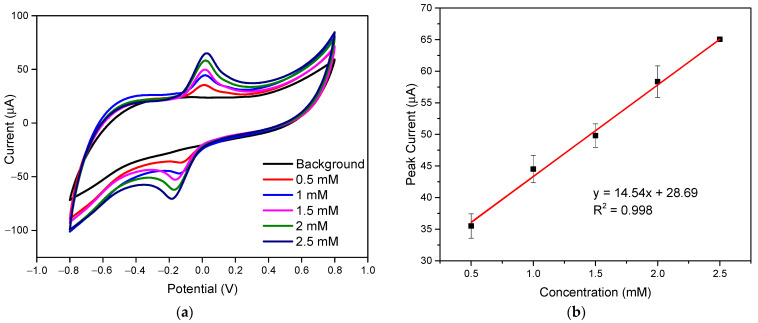
(**a**) CV responses of ferricyanide solutions with varying concentrations; and (**b**) oxidation current peaks vs. concentration. Scan rate was 50 mV s^−1^.

Before developing the glucose biosensor with LIGE, validating the LIGE sensor towards electrochemical sensing was necessary. The ferri/ferrocyanide (Fe(CN)_6_^3−/4−^) redox couple is one of the most widely used electron mediators for electrochemical reactions [39]. The performance of an electrochemical sensor towards an electron mediator was considered most relevant to general biochemical sensing applications. Thus, the electrochemical efficacy of the LIGE sensor was evaluated using cyclic voltammetry responses in different concentrations of ferricyanide redox mediator (K_3_[Fe(CN)_6_]), as shown in Figure 3a. As seen from Figure 3a, the oxidation peaks’ current increased from 35.495 to 65.043 µA when the ferricyanide concentration ranged from 0.5 to 2.5 mM. The oxidation peak current showed an excellent linear relationship with different ferricyanide concentrations, as shown in Figure 3b. The linear regression equation was $y=14.54x+28.69\;\left(R^{2}=0.998\right)$, where *y* and *x* are the height of oxidation peak current (µA) and (K_3_[Fe(CN)_6_]) concentration (mM), respectively. The fabricated LIGE provided a favorable response for varying ferricyanide concentrations, indicating excellent electrocatalytic properties. Moreover, the reproducibility of all CV responses was within 5% RSD (relative standard deviation) (*n* = 4). These results demonstrated the remarkable electrocatalytic response of the fabricated LIGE sensor.

### 3.2. Characterization of GOx/Chitosan Immobilized LIGE

**Figure 4:**
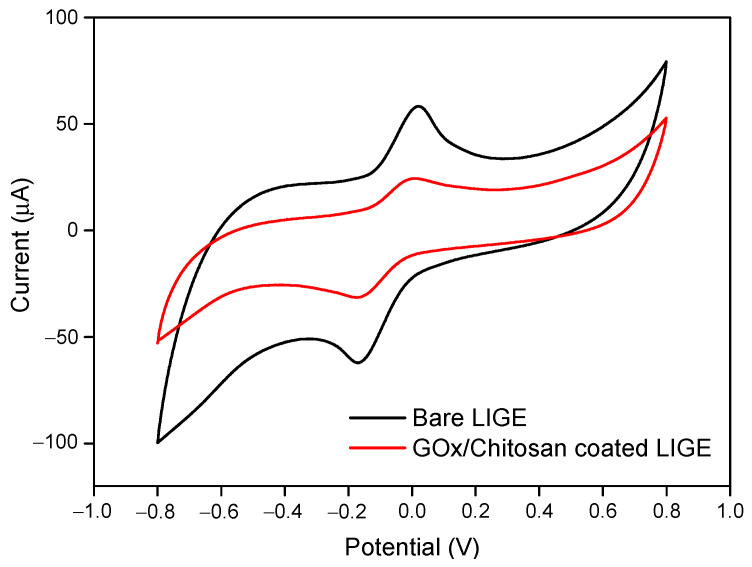
Cyclic voltammograms on bare LIGE and GOx/chitosan-modified LIGE in the presence of 2 mM potassium ferricyanide.

Cyclic voltammetry measurement was performed to confirm the LIGE immobilization with GOx/Chitosan. Figure 4 shows the cyclic voltammograms of potassium ferricyanide at bare LIGE and GOx/chitosan composite-modified LIGE. It can be seen that after the immobilization of GOx/chitosan composite onto the LIGE surface, the peak current decreased to 24.325 from 58.336 µA of the bare LIGE. The electron transfer kinetics of [Fe(CN)6]^4−^/[Fe(CN)6]^3−^ is significantly hindered after the LIGE surface was modified with GOx/chitosan. This result confirmed that the GOx/chitosan was successfully immobilized on the LIGE surface.

### 3.3. Amperometric Detection of Glucose by the Proposed LIGE

**Figure 5:**
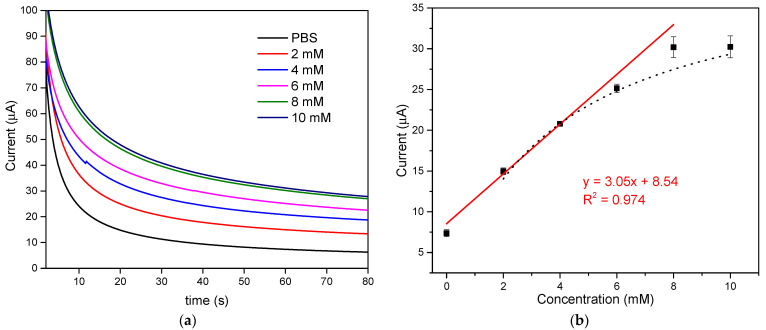
(**a**) Chronoamperometry response with different concentrations of glucose; (**b**) the relationship between the glucose concentration and the chronoamperometric current response at 60 s.

The chronoamperometry technique was employed to detect glucose using GOx/Chitosan coated LIGE sensor at a constant oxidation potential of +0.8 V. Figure 5a depicts the chronoamperometric responses of the LIGE biosensor with glucose concentrations ranging from 0 to 10 mM. The current response increased with increasing glucose concentrations. The steady-state current response at 60 s was chosen for the detection of glucose concentration. The amperometric current response of the LIGE biosensor exhibited a linear relationship with the glucose concentrations ranging from 0 to 8 mM, and the current began to level off at a glucose concentration higher than 8 mM as shown in Figure 5b. The linear regression equation was y=3.05x+8.54, with a coefficient of determination *R*^2^ = 0.97 and a sensitivity of 43.15 µA mM^−1^ cm^−2^. The limit of detection was calculated according to the 3s_a_/b criterion, where b was the slope of the calibration curve, and s_a_ was the estimated standard deviation of the y-intercepts of the regression line [3]. The detection limit calculated was 0.431 mM. As seen from Figure 5b, the linear part of the calibration curve includes the normal glucose levels (4.4 to 6.6 mM) in the human blood. Thus, this study could offer a simple approach for the clinical glucose measurement with a disposable LIGE-based biosensor. The performance of the proposed biosensor was compared with other reported glucose biosensors, as shown in Table 1. The developed LIGE-based enzymatic glucose biosensor exhibited good analytical characteristics towards glucose detection such as good linearity and high sensitivity. Moreover, the fabrication and detection procedures of the proposed LIGE-based biosensor were also simple, rapid, and cost-effective.

### 3.4. Michaelis–Menten Kinetics

The maximum response current ($I_{max}$) and the apparent Michaelis–Menten constant ($K_{m}^{\mathrm{app}}$) were used to analyze the relationship between chronoamperometric signals and enzymatic reaction. As shown in Figure 5b, when glucose concentration exceeds 6 mM, a response plateau was observed with the characteristic of Michaelis–Menten kinetic mechanism. From the calibration plot (Figure 5b dotted line), the current response showed hyperbolic dependence on glucose concentration and was in good agreement with Michaelis–Menten kinetics [45]. The kinetic parameters, the maximum current generated during the enzymatic reaction ($I_{max}$) and the apparent Michaelis constant ($K_{m}^{\mathrm{app}}$) are the corresponding a and b parameters of hyperbolic function y=ax/(b+x) [46]. The apparent Michaelis–Menten constant ($K_{m}^{\mathrm{app}}$) is an indication of enzymatic mimics–substrate kinetics. From the hyperbolic calibration plot (Figure 5b dotted line), the $I_{max}$ and Michaelis constant $K_{m}^{\mathrm{app}}$ were 40.34 µA and 3.75 mM, respectively. The value of $K_{m}^{\mathrm{app}}$ is consistent with the reported value ($K_{m}^{\mathrm{app}}=3.84\;\mathrm{mM}$) for other GOx immobilized on the chitosan complex over triangular silver nanoprisms/platinum biosensor [47].

### 3.5. Optimization of Applied Potential and Buffer pH

**Figure 6:**
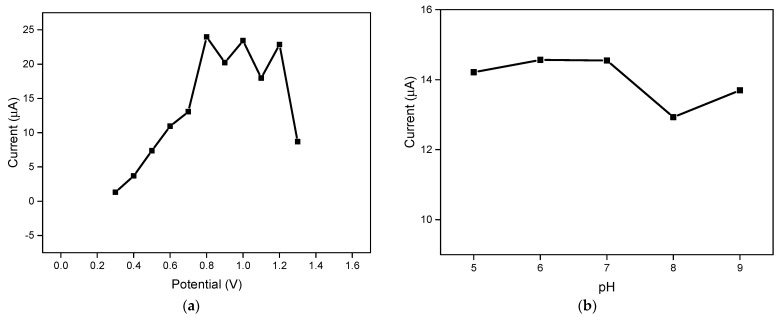
(**a**) Chronoamperometry response at 60 s in different applied potential with 5 mM glucose; and (**b**) Chronoamperometry response at 60 s in different buffer pH with 2 mM glucose.

Chronoamperometry measurements were used to determine the optimal applied potential and pH for glucose detection with the developed enzymatic LIGE biosensor. Figure 6a shows the chronoamperometric response of the LIGE biosensor at 60 s with different applied potential values ranging from 0.3 to 1.3 V. The results showed that the current increased with increasing applied potential from 0.3 to 0.8 V and currents tended to level off when the potential increased beyond 0.8 V. Thus, 0.8 V was selected as the optimized potential for amperometric glucose detection. Figure 6b illustrates the chronoamperometry current response of the biosensor as a function of the pH of PBS containing 2 mM glucose. The current responses at pH 5, pH 6, and pH 7 were almost similar. Considering the pH of a physiological buffer, pH 7 was chosen for the glucose detection experiments.

### 3.6. Interference Study

**Figure 7:**
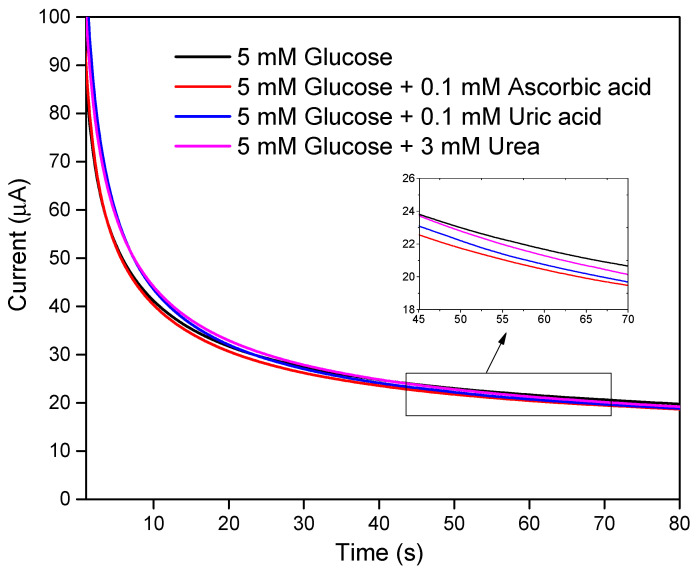
Chronoamperograms of 5 mM glucose with/without interferences. The inset shows the zoomed part of the result from 45 to 70 s.

The developed LIGE-based enzymatic biosensor was evaluated with possible interferences by comparing the chronoamperometric responses before and after adding some interferents such as ascorbic acid (0.1 mM), uric acid (0.1 mM), and urea (3 mM) in 5 mM glucose. As shown in Figure 7, the chronoamperometric current responses for glucose without and with interferents showed practically no interference. The LIGE was modified with GOx, which is the standard enzyme for biosensors and it has relatively higher selectivity for glucose [48]. Hence, the LIGE biosensor was suggested to possess good selectivity due to the specificity of the GOx enzyme.

### 3.7. Stability and Reproducibility of Biosensor

The stability of the developed GOx/chitosan-modified LIGE biosensor was evaluated by measuring the amperometric current response in the presence of 5 mM glucose over 25 days stored at 4 °C in a refrigerator. The biosensor exhibited ~90% stability for 10 days, and the response remained approximately 72–85% after 10 days. The reproducibility of the developed biosensor was assessed from the current response of different biosensors prepared independently. In this work, all the measurements were taken from at least three independent sensors (*n* ≥ 3), and the reproducible signals were obtained with the RSD less than 6%.

## 4. Conclusions

We developed a simple laser-induced graphene-based enzymatic biosensor for glucose detection. The proposed detection strategy could offer an easy and low-cost route to mass-produce sensitive biosensing electrodes. The chronoamperometric measurements successfully detected the glucose over a linear range from 0 to 8 mM with a detection limit of 0.431 mM. The biosensor response was not affected by interfering compounds (ascorbic acid, uric acid and urea) and demonstrated the high specificity and selectivity of this LIGE biosensor in glucose detection. The proposed LIGE biosensor holds excellent promise in point-of-care diagnosis. Our future study aims to validate the biosensor response in human blood samples for real-life applications.

## Figures and Tables

**Table 1:** Comparison of the analytical performance of glucose biosensors.

Glucose Biosensor ^a^	Sensitivity (µA mM^−1^ cm^−2^)	Linear Range (mM)	LOD (µM)	Reference
GOx/Chitosan-modified LIGE	43.15	0–8	431	This work
GC/MWCNT/Fe_3_O_4_/PDA–GOx	5.04	2–20	2.25	[40]
LSG/PBSE/PtNPs/GOx	12.64	0.005–3.2	2.57	[41]
MoS_2_/Chitosan/GOx-Gelatin/PGE	0.8 (µA mM^−1^)	0.01–0.8	3.18	[42]
CPE/GOx-SiO_2_/Lig	0.78	0.5–9	145	[19]
Au–Cys–GA–Gox	2.65	1.5–7	940	[43]
PPy/GOD/SPCE	0.21	0–5	-	[44]

^a^ GC—glassy carbon electrode; MWCNT—multi-walled carbon nanotubes; Fe_3_O_4_/PDA—magnetite/polydopamine; LSG—laser-scribed graphene; PBSE—pyrenebutanoic acid–succinimide ester; PtNPs—platinum nanoparticles; MoS_2_—molybdenum disulfide; PGE—pencil graphite electrode; CPE—carbon paste electrode; SiO_2_/Lig—silica/lignin; Cys—cysteine; GA—glutaraldehyde; PPy—polypyrrole; SPCE—screen-printed carbon electrodes; GOD/GOx—Glucose oxidase.
